# The diagnostic and predictive value of ultrasonography in congenital diaphragmatic hernia

**DOI:** 10.3389/fped.2025.1726224

**Published:** 2026-01-23

**Authors:** Qin Liu, Hongyan Ren, Mingxue Wang, Zhong Feng, Lishuang Ma

**Affiliations:** 1 Department of Ultrasound, Capital Center for Children’s Health, Capital Medical University, Beijing, China; 2 Department of Neonatal Surgery, Capital Center for Children’s Health, Capital Medical University, Beijing, China; 3 Graduate School of Chinese Academy of Medical Sciences & Peking Union Medical College, Beijing, China

**Keywords:** congenital diaphragmatic hernia, infants, neonatal, prognostic prediction, ultrasound

## Abstract

**Objective:**

The current diagnostic and prognostic assessment of congenital diaphragmatic hernia (CDH) in neonates remains challenging. This study aimed to evaluate the utility of neonatal ultrasonography in the diagnosis and prognostic prediction of CDH in infants.

**Materials and methods:**

A retrospective analysis was conducted on clinical data from 152 infants diagnosed with CDH and admitted to the Department of Neonatal Surgery at Children's Hospital between 2017 and 2023. The cohort included 86 (56.6%) males and 66 (43.4%) females. Multivariate logistic regression was employed to identify factors associated with CDH prognosis. Receiver operating characteristic (ROC) curve analysis was performed to assess the predictive value of significant ultrasonographic indicators.

**Results:**

Multivariate logistic regression identified four factors as significant predictors of mortality: diaphragmatic defect length >4 cm [odds ratio [OR] = 2.41, 95% confidence interval [CI]: 1.08–10.58], the presence of hepatic herniation (OR = 2.61, 95% CI: 1.16–5.87), absence of a hernial sac (OR = 4.86, 95% CI: 2.00–11.76), and concomitant lung ultrasound abnormalities (OR = 10.86, 95% CI: 1.28–21.85). The combination of these four parameters demonstrated strong predictive performance for mortality, with an area under the ROC curve of 0.860 (95% CI: 0.786–0.935).

**Conclusion:**

Diaphragmatic defect length, hepatic herniation, hernial sac status, and lung ultrasound findings serve as valuable prognostic indicators in infants with CDH. Integrating these four parameters enhances prognostic accuracy and may support clinical decision-making.

## 1 Introduction

Congenital diaphragmatic hernia (CDH) is a congenital anomaly resulting from embryonic maldevelopment of the diaphragm (1, 2). This defect permits the herniation of abdominal organs into the thoracic cavity, inducing a series of pathophysiological changes. With an incidence of approximately 1 in 3,000 to 1 in 5,000 live births, CDH remains one of the most challenging conditions in pediatric surgery (3). Affected infants often present with concomitant pulmonary hypoplasia and pulmonary hypertension (4). Furthermore, 10%–40% of cases are associated with severe malformations in other systems-such as congenital heart defects-which significantly contribute to the wide variability in disease severity and postnatal outcomes (5). While mild cases may achieve full survival, severe cases exhibit mortality rates exceeding 70% (6). Consistent with this, Cruz-Martínez et al. reported a high neonatal mortality rate of 68.1% (98/144) among CDH cases in Latin American countries (7).

Accurate risk stratification following the prenatal diagnosis of CDH is critically important for both clinicians and parents, as it informs decisions regarding pregnancy continuation, timing of delivery, potential prenatal interventions, and planning for postnatal resuscitation. It also aids in determining the appropriate level of neonatal care, facilitates timely transfer to specialized pediatric centers, and enhances clinical vigilance among healthcare providers and families, thereby optimizing the timing of life-saving treatments. Previous studies have established the important role of ultrasound in the diagnosis of CDH (8–10). For instance, Werneck Britto et al. demonstrated that two-dimensional ultrasound measurements had predictive accuracy comparable to that of magnetic resonance imaging for assessing neonatal outcomes (9). However, existing evidence on sonographic risk factors for infant CDH remains inconsistent. Moreover, while prognostic evaluations have predominantly focused on the prenatal period, there is a relative scarcity of studies on postnatal ultrasound assessment, limiting the ability of neonatal surgeons to develop individualized management strategies for affected infants (5). In China, research on the prognostic evaluation of CDH is still in its early stages, and few studies based on Chinese neonates have reported the value of ultrasound in both diagnosis and outcome prediction. For instance, in 2014, Zhou Lei et al., based on ultrasound imaging of 8 fetuses with congenital diaphragmatic hernia (CDH), suggested that comprehensive and detailed prenatal ultrasound examinations could provide a clearer diagnosis of fetal CDH and hold important clinical significance for prognostic assessment of the disease (11). In 2015, He Qiuming et al., based on the treatment of 14 CDH infants, found that thoracoscopic surgery yielded satisfactory outcomes in the management of neonatal CDH (12). In 2022, Wang Xinyin et al., based on a study of 57 CDH infants, demonstrated that fetal MRI signs and predictive scoring models could effectively identify the presence or absence of a hernia sac in infants with congenital diaphragmatic hernia, thereby holding certain clinical significance (13).

Therefore, utilizing data from the Neonatal Surgery Department of Children's Hospital, this study aimed to explore the role of neonatal ultrasound in the diagnosis and prognostic assessment of CDH among infants in China.

## 2 Methods

### 2.1 Study participants

This study retrospectively enrolled 154 infants with CDH admitted to the Department of Neonatal Surgery at Children's Hospital between January 2017 and December 2023. Two infants were excluded due to missing data on initial diagnosis and associated congenital anomalies. Thus, 152 infants [86 boys [56.6%] and 66 girls [43.4%]] were included in the final analysis. Written informed consent was obtained from the parents or guardians of all participants. The study protocol was approved by the Ethics Committee of the Capital Institute of Pediatrics (approval no. SHERLLM2022009).

### 2.2 Testing

**Figure 1:**
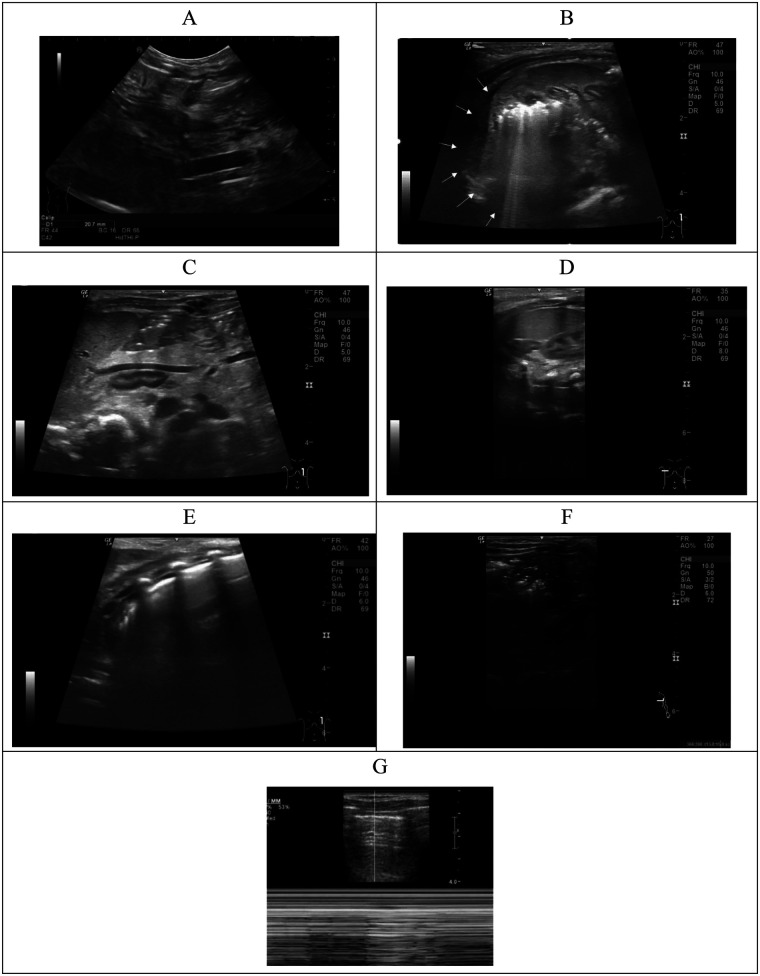
Ultrasonic diagnosis in children with CDH. **(A)** Measurement of diaphragmatic defect length (calipers); **(B)** presence of a hernia sac (arrowhead); **(C)** herniated abdominal contents, including the spleen, left adrenal gland, stomach, and intestinal loops, without liver involvement (infra-hepatic type); **(D)** herniated contents involving partial liver **(L)** protrusion into the thoracic cavity (suprahepatic type); **(E)** lung ultrasound showing interstitial edema, characterized by multiple, confluent B-lines; **(F)** atelectasis with the presence of an air bronchogram sign; **(G)** pneumothorax demonstrated on M-mode ultrasound by the stratosphere sign (parallel lines) and the absence of lung sliding.

All examinations were performed using a GE LOGIQ 9 full-digital color Doppler ultrasound system. Diaphragmatic and abdominal assessments were conducted using an L9 linear array probe at a frequency of 9 MHz. Infants were placed in a supine or lateral decubitus position. The entire diaphragmatic cross-section was scanned via the upper abdomen to evaluate the position, morphology, echogenicity, and mobility of the diaphragmatic margin and adjacent lung base. Thoracic back scanning was performed to assess lung development, herniated contents, and cardiac position. The coronal view of the upper abdomen or flank was used to examine the position and morphology of the liver, kidneys, and spleen. Hepatic herniation was classified as suprahepatic if part or all of the liver herniated into the thoracic cavity, and infrahepatic if no liver herniation was present. Representative ultrasound images are provided in Figure 1.

The lung examination was performed in accordance with the “guideline on lung ultrasound to diagnose pulmonary diseases in newborn infants” (14), as detailed below: (1) Probe selection: Linear array probe with a frequency of 9–12 MHz. (2) Lung division: The lungs are generally divided into three regions-anterior, lateral, and posterior-using the anterior and posterior axillary lines as boundaries, resulting in six regions for both lungs. To avoid missing areas during examination, each lung may be further divided into upper and lower fields using the nipple line as a reference, thus creating a total of 12 regions. (3) Positioning: During the examination, the infant may be placed in supine, lateral, or prone positions, and each lung region is scanned separately. (4) Scanning method: The probe should be positioned perpendicular to the ribs (longitudinal scan) and parallel to the ribs (transverse scan: scanning along each intercostal space). The longitudinal scan is the most important and commonly used method, with perpendicular alignment of the probe to the ribs being crucial for accurate and reliable examination. (5) Neonatal pulmonary atelectasis (NPA): Ultrasound has definitive diagnostic value for NPA, primarily based on the following criteria: (a) Pulmonary consolidation with bronchial air sign: In severe cases, parallel bronchial air signs or bronchial fluid signs may be observed; (b) Dynamic bronchial air sign: In early stages of severe or extensive NPA, dynamic bronchial air signs are visible under real-time ultrasound; in later stages, these signs often disappear; (c) In extensive NPA with severe consolidation, the edges of the consolidated area are typically clear, regular, and sharp. In small, localized NPA, the boundary between the consolidated area and surrounding lung tissue may be indistinct; (d) Lung pulse and lung sliding: In early stages of severe or extensive atelectasis, lung pulse is often visible under real-time ultrasound, while lung sliding is usually absent. In small, localized atelectasis, lung pulse may not be obvious, and lung sliding may still be present; (e) Abnormal pleural line and disappearance of A-lines in the consolidated area, while non-consolidated areas may retain these features; (f) Color Doppler ultrasound may reveal pulmonary blood flow (spectral signal) in the consolidated area, which serves as the physiological basis for the recovery of atelectatic lung tissue. In advanced stages, pulmonary blood flow may disappear. (6) Pneumothorax: Ultrasound is accurate and reliable for diagnosing pneumothorax, primarily based on the following: (a) Absence of lung sliding under real-time ultrasound: This is the most important sign for diagnosing pneumothorax; its presence essentially rules out pneumothorax; (b) Presence of pleural line and A-lines: Their absence largely excludes pneumothorax; (c) Absence of B-lines: Their presence also largely rules out pneumothorax; (d) Definitive lung point: A specific sign for mild to moderate pneumothorax; (e) Stratosphere sign in M-mode ultrasound at the site of gas accumulation. (7) Pulmonary interstitial edema: (a) Discrete B-lines: Continuous and symmetrical, with a smooth, uniform, and intact pleural line. Based on spacing (mm), they are classified as B3 and B7 lines. B3 lines suggest ground-glass opacities in lung tissue, indicating alveolar pulmonary edema; B7 lines suggest thickening of the interlobular septa, indicating possible interstitial pulmonary edema; (b) Coalescent B-lines: B-lines become densely packed and merge together, with an uneven or discontinuous pleural line and reduced or absent lung sliding, indicating increased pulmonary edema and aggravated alveolar edema.

To ensure accuracy, all scans and image interpretations were performed by pediatric ultrasound specialists with at least five years of experience. Infants were examined in a quiet state, preferably in a prone position, to obtain stable images. Each infant underwent at least three repeated scans at the same anatomical site.

### 2.3 Definitions

Study participants were categorized into death or survival groups based on clinical outcome. Hepatic position was classified as herniated or non-herniated. Diaphragmatic defects were categorized by location as right-sided, left-sided, or bilateral. The presence or absence of a hernia sac was also recorded. The maximum diaphragmatic defect length was measured ultrasonographically (Considering the timeliness of clinical decision-making and data completeness, we selected ultrasound data for analysis rather than intraoperative measurements, as this approach better aligns with the clinical predictive logic and practical application scenarios of the study) and dichotomized as ≤4 cm or >4 cm based on receiver operating characteristic (ROC) analysis, which identified 4 cm as the optimal cutoff for predicting mortality. Lung ultrasound abnormalities were defined as the presence of pneumothorax, interstitial edema, or pulmonary atelectasis. A hernia sac was considered present if any of the following sonographic features were observed: (a) a curved crescent-shaped line posterior to the lung or at the tip of the herniated contents; (b) wrapped appearance of herniated contents with minimal compression of the heart and mediastinum; (c) cystic pleural fluid collection overlying the lung; or (d) cystic ascites beneath the lung. Both the diaphragmatic defect size and the presence of a hernia sac, as determined by ultrasound, were confirmed intraoperatively.

### 2.4 Other variables

Birth weight, birth length, mode of delivery (cesarean vs. vaginal), and gestational age were obtained from birth medical records. Data regarding the initial diagnosis of CDH and the presence of associated congenital anomalies were extracted from hospital medical records.

### 2.5 Statistical analysis

Continuous variables are expressed as mean ± standard deviation and compared using the Student's *t*-test. Categorical variables are presented as numbers (percentages) and compared using the chi-square test. Multivariable logistic regression was employed to assess the associations between CDH outcomes and potential influencing factors, including diaphragmatic defect length, hepatic herniation, hernia sac status, and lung ultrasound abnormalities. ROC analysis was used to evaluate the area under the curve (AUC) of these parameters, both individually and in combination, for predicting CDH outcomes. All analyses were performed using SAS version 9.4 (SAS Institute, Cary, NC, USA). A two-sided *p*-value < 0.05 was considered statistically significant.

## 3 Results

### 3.1 Characteristics of participants

The baseline characteristics of the 152 enrolled infants are summarized in Table 1. The cohort had a mean birth length of 49.6 ± 4.1 cm and a mean birth weight of 3.1 ± 0.9 kg. Of these, 40 infants (26.3%) were delivered by cesarean section, and 31 (20.4%) had associated congenital anomalies. Hepatic herniation was present in 46 infants (30.3%), and 61 (40.1%) were initially diagnosed before 24 gestational weeks. Lung ultrasound abnormalities were identified in 108 infants (71.1%).

**Table 1:** Characteristics of participants.

Characteristics	Total	Healed and without complications	Healed and with complications	Recurrence	Death	*P*
*N*	152	42	72	7	31	
Birth length, cm	49.6 ± 4.1	50.7 ± 6.0	49.4 ± 3.0	49.6 ± 2.1	48.5 ± 3.1	0.164
Birth weight, kg	3.1 ± 0.9	3.3 ± 1.2	3.1 ± 0.8	3.1 ± 0.3	2.9 ± 0.7	0.198
Hernia area, cm^2^	14.5 ± 14.9	14.3 ± 16.5	13.1 ± 12.5	18.5 ± 9.8	17.2 ± 18.5	0.550
Diaphragmatic defect length, cm	3.7 ± 1.3	3.4 ± 1.2	3.6 ± 1.3	4.6 ± 1.2	4.1 ± 1.5	0.026
Sex, *n* (%)						0.770
Boys	86 (56.6)	22 (25.6)	40 (46.5)	4 (4.7)	20 (23.3)	
Girls	66 (43.4)	20 (30.3)	32 (48.5)	3 (4.5)	11 (16.7)	
Mode of delivery, *n* (%)						0.300
Caesarean birth	40 (26.3)	13 (32.5)	21 (52.5)	2 (5.0)	4 (10.0)	
Natural birth	112 (73.7)	29 (25.9)	51 (45.5)	5 (4.5)	27 (24.1)	
Gestational week						0.351
Mature	126 (82.9)	37 (29.4)	61 (48.4)	5 (4.0)	23 (18.3)	
Premature	26 (17.1)	5 (19.2)	11 (42.3)	2 (7.7)	8 (30.8)	
Associated congenital anomalies, *n* (%)						<0.001
Yes	31 (20.4)	6 (19.4)	8 (25.8)	2 (6.5)	15 (48.4)	
No	121 (79.6)	36 (29.8)	64 (52.9)	5 (4.1)	16 (13.2)	
Hepatic hernia, *n* (%)						<0.001
Yes	47 (30.9)	7 (14.9)	17 (36.2)	3 (6.4)	20 (42.6)	
No	105 (69.1)	35 (33.3)	55 (52.4)	4 (3.8)	11 (10.5)	
First diagnose, *n* (%)						0.018
≤24 weeks	61 (40.1)	13 (21.3)	25 (41.0)	3 (4.9)	20 (32.8)	
>24 weeks	91 (59.9)	29 (31.9)	47 (51.6)	4 (4.4)	11 (12.1)	
Herniation sac						<0.001
Yes	84 (55.3)	27 (32.1)	47 (56.0)	2 (2.4)	8 (9.5)	
No	68 (44.7)	15 (22.1)	25 (36.8)	5 (7.4)	23 (33.8)	
Lung ultrasound abnormalities						<0.001
Yes	108 (71.1)	0 (0.0)	72 (66.7)	6 (5.6)	30 (27.8)	
No	44 (28.9)	42 (95.5)	0 (0.0)	1 (2.3)	1 (2.3)	
Hernia location, *n* (%)						0.805
Left	114 (75.0)	31 (27.2)	56 (49.1)	6 (5.3)	21 (18.4)	
Right	37 (24.3)	11 (29.7)	15 (40.5)	1 (2.7)	10 (27.0)	
Both	1 (0.7)	0 (0.0)	1 (100.0)	0 (0.0)	0 (0.0)	

Among the 152 infants with CDH, 31 resulted in death, while 121 survived. Of the survivors, 42 recovered without complications, 72 recovered with complications, and 7 experienced recurrence. The diaphragmatic defect length was significantly greater in the death/recurrence group compared to the group that healed without complications (*P* = 0.026). Mortality was significantly higher in infants with associated congenital anomalies than in those without (48.4% vs. 13.2%). Similarly, a higher proportion of deaths was observed among infants without a hernia sac (33.8% vs. 9.5% with a sac), those with hepatic herniation (42.6% vs. 10.5% without), those diagnosed before 24 gestational weeks (32.8% vs. 12.1% diagnosed later), and those with lung ultrasound abnormalities (27.8% vs. 2.3% without) (Table 1).

### 3.2 Factors associated with mortality

As shown in Table 2, multivariable logistic regression identified four factors significantly associated with CDH mortality: a diaphragmatic defect length >4 cm (OR = 2.41, 95% CI: 1.08–10.58), the presence of hepatic herniation (OR = 2.61, 95% CI: 1.16–5.87), the absence of a hernia sac (OR = 4.86, 95% CI: 2.00–11.76), and the presence of lung ultrasound abnormalities (OR = 10.86, 95% CI: 1.28–21.85).

**Table 2:** Logistic regression analysis of congenital diaphragmatic hernia death.

Predictors	*β*	*OR*	*95%CI*	*P*
Diaphragmatic defect length				
≤4 cm		1.00		
>4 cm	0.879	2.41	1.08–10.58	0.044
Hepatic hernia				
No		1.00		
Yes	0.958	2.61	1.16–5.87	0.021
Herniation sac				
Yes		1.00		
No	1.580	4.86	2.00–11.76	<0.001
Lung ultrasound abnormalities				
No		1.00		
Yes	2.385	10.86	1.28–21.85	0.029

Sex, birth length, birth length, hernia location, mode of delivery, gestational week, associated congenital anomalies, first diagnose time, and all variables listed in the table were introduced into logistic regression models.

### 3.3 Predictive value of ultrasound parameters

**Figure 2:**
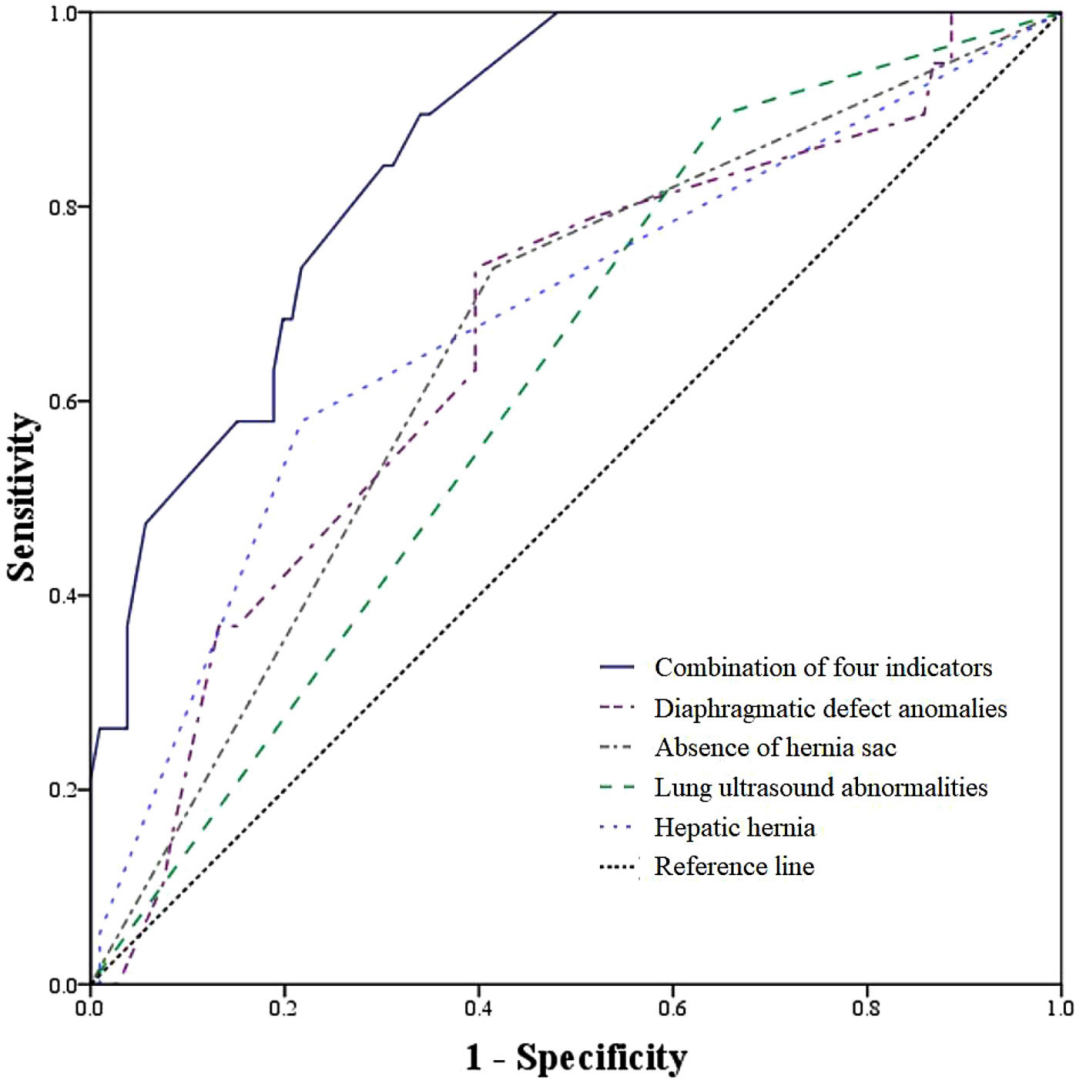
ROC analysis for predicting death according to diaphragmatic defect length, hepatic hernia, lung ultrasound abnormalities, absence of hernia sac, and combination of the four indicators.

Receiver operating characteristic (ROC) analysis was performed to evaluate the predictive performance of individual and combined parameters for CDH mortality. The area under the curve (AUC) for hepatic herniation was 0.684 (95% CI: 0.544–0.824), for diaphragmatic defect length was 0.665 (95% CI: 0.533–0.797), for lung ultrasound abnormalities was 0.662 (95% CI: 0.569–0.755), and for the absence of a hernia sac was 0.622 (95% CI: 0.499–0.745). Notably, the combination of all four parameters demonstrated a substantially higher predictive ability, with an AUC of 0.860 (95% CI: 0.786–0.935) (Figure 2).

## 4 Discussion

In this study, the case fatality rate among infants with CDH was 20.4% (31/152). Multivariable analysis identified lung ultrasound abnormalities, hepatic herniation, absence of a hernia sac, and a diaphragmatic defect length >4 cm as independent risk factors for mortality. Furthermore, the combination of these four parameters demonstrated a superior predictive value for prognostic assessment compared to any single indicator.

Previous studies have reported varying mortality rates for CDH. Colvin et al., using data from Western Australia (1991–2002), documented a mortality rate of 29.3% (34/116) (15). Similarly, Grover et al. reported a rate of 29.2% (167/572) from 27 hospitals in Chicago (2010–2013) (16), while a large-sample study by Hagadorn et al. indicated a rate exceeding 34.0% (1064/3,123) (17). Although the mortality rate in our cohort is somewhat lower, the finding that over one-fifth of infants succumbed to CDH underscores its status as a significant clinical challenge in neonatology.

Our results confirm hepatic herniation as a significant predictor of mortality. This is consistent with existing literature; Hedrick et al. reported approximately 55% mortality and an 80% rate of ECMO requirement among neonates with hepatic herniation (18). Kitano et al. further noted mortality rates ranging from 53% to 90% for such cases (19). Notably, Hedrick et al. observed a stark contrast in mortality between neonates with left hepatic herniation (65%) and those without (7%) (20), reinforcing the critical prognostic implication of liver position.

The size of the diaphragmatic defect serves as a direct morphological indicator of diaphragmatic developmental failure. Our finding that a defect length >4 cm is a risk factor for death aligns with a previous study of 140 children, which associated larger defects with poorer prognoses (21). Furthermore, the association between comorbid lung pathology, as detected by ultrasound, and increased mortality risk is consistent with established knowledge (22) and is corroborated by our results.

The hernia sac, believed to result from the persistence of membranous tissue after the closure of the pleuroperitoneal canal (16), was also a significant prognostic factor. Hagadorn et al. found a markedly higher survival rate in children with a hernia sac (94.4% vs. 67.3%, *P* = 0.03) (17), a finding echoed by Gentili et al. (100% vs. 32%, *P* < 0.01) (23). Our study corroborates the absence of a hernia sac as an independent risk factor. A proposed mechanism is that the sac may partially restrict the herniation of abdominal contents into the thorax, thereby mitigating compression on the lungs and mediastinum (24, 25).

While several prenatal scoring systems exist for predicting CDH prognosis (20), ur study focuses on postnatal sonographic indicators. We demonstrated that the combination of four readily assessable ultrasound parameters-diaphragmatic defect length, hepatic herniation, hernia sac status, and lung abnormalities-provides a valuable and significantly more accurate prognostic model for infants after birth.

This study has several limitations. First, the sample size of 152 infants may have limited statistical power and precluded meaningful subgroup analyses. Second, despite adjusting for multiple confounders, residual confounding from unmeasured factors cannot be ruled out. Third, the cross-sectional design of the analysis necessitates caution in inferring causality. Fourth, as a single-center study, the generalizability of our findings to other populations may be limited. Fifth, Specific data on ECMO use were incomplete, thus precluding its reliable inclusion as an analytical endpoint in the current multivariate model. Future studies should incorporate ECMO or a composite clinical deterioration endpoint into the analysis. Sixth, due to the time period during which this study was conducted, we did not employ the more standardized Lung Ultrasound (LUS) score-which has been recommended in recent years-as an indicator for assessing lung abnormalities. Future prospective studies should adopt such scoring systems for validation. Finally, the study did not incorporate prenatal ultrasound indices, such as the lung-to-head ratio, which could provide a more comprehensive prognostic picture.

## 5 Conclusion

The combination of diaphragmatic defect length, hepatic herniation, hernia sac status, and lung ultrasound findings provides an accurate tool for predicting mortality risk in infants with CDH. This integrated postnatal assessment allows for early and precise severity stratification, enabling clinicians to tailor therapeutic interventions promptly. The application of this model holds the potential to improve clinical outcomes and enhance the survival rate and quality of life for affected infants.

## Data Availability

The datasets presented in this article are not readily available because data are available from the corresponding author upon reasonable request. Requests to access the datasets should be directed to Qin Liu, 52306551@qq.com.
